# Prognostic Value of N6-Methyladenosine-Related lncRNAs in Early-Stage Colorectal Cancer: Association With Immune Cell Infiltration and Chemotherapeutic Drug Sensitivity

**DOI:** 10.3389/fmolb.2021.724889

**Published:** 2021-10-12

**Authors:** Zhizhong Xiong, Xianzhe Li, Shi Yin, Minghao Xie, Chaobin Mao, Fengxiang Zhang, Huaxian Chen, Longyang Jin, Lei Lian

**Affiliations:** ^1^ Department of Gastrointestinal Surgery, The Sixth Affiliated Hospital, Sun Yat-Sen University, Guangzhou, China; ^2^ Guangdong Provincial Key Laboratory of Colorectal and Pelvic Floor Diseases, Guangdong Institute of Gastroenterology, The Sixth Affiliated Hospital of Sun Yat-Sen University, Guangzhou, China

**Keywords:** early-stage colorectal cancer, long non-coding RNA, N6-methyladenosine, nomogram, prognosis

## Abstract

**Purpose:** Accumulating evidence indicates that N6-methyladenosine-related long non-coding RNAs (m6A-related lncRNAs) play a crucial role in the occurrence and development of several cancers. We aimed to explore the potential role of m6A-related lncRNA signatures in predicting prognosis for early-stage (stages I and II) colorectal cancer (CRC).

**Methods:** m6A-related lncRNA data were obtained from The Cancer Genome Atlas. Univariate Cox regression analysis was used to screen for prognostic m6A-related lncRNAs. Immune characteristics were analyzed in different subgroups created via unsupervised clustering analysis. Next, patients were randomly divided into training and test cohorts. In the training cohort, least absolute shrinkage and selection operator (LASSO) regression was performed to establish a prognostic model. The predictive value of the signature was evaluated in the training and test cohorts. Drug sensitivity was also examined.

**Results:** A total of 1,478 m6A-related lncRNAs were identified. Two subgroups were created based on the expression of seven prognostic m6A-related lncRNAs. Prognosis was worse for cluster 1 than for cluster 2, and cluster 1 was characterized by increased numbers of M2 macrophages, decreased numbers of memory B cells, and higher expression of checkpoint genes when compared with cluster 2. Five m6A-related lncRNAs were selected to establish a risk prediction signature via LASSO regression. The 3 years overall survival (OS) was higher in the low-risk group than in the high-risk group. The area under the curve at 1, 2, and 3 years was 0.929, 0.954, and 0.841 in the training cohort and 0.664, 0.760, and 0.754 in the test cohort, respectively. Multivariate Cox regression analysis suggests that the risk score was an independent predictor of OS in both the training and test cohorts. A prognostic nomogram based on the five m6A-related lncRNAs and their clinical features was built and verified. The high-risk group was more sensitive to chemotherapeutic drugs (camptothecin and cisplatin) than the low-risk group.

**Conclusion:** We identified two molecular subgroups of early-stage CRC with unique immune features based on seven prognostic m6A-related lncRNAs. Subsequent analyses demonstrated the usefulness of a five m6A-related lncRNA signature as a potential indicator of prognosis in patients with early-stage CRC.

## Introduction

Colorectal cancer (CRC) is one of the most common gastrointestinal malignancies and is among the main causes of cancer-related deaths. Although the 5 years survival rate for early-stage CRC (stage I and stage II) can reach 90%, these patients are still at risk of recurrence, which is often fatal (Siegel et al., 2019; Walker et al., 2020). Whether chemotherapy is necessary for patients with early-stage CRC depends mainly on known clinical and pathological risk factors, such as microsatellite instability (MSI) status and bowel obstruction (Manfredi et al., 2006; Kannarkatt et al., 2017). However, these high-risk factors cannot distinguish between patients with poor prognosis and those who benefit from chemotherapy (Dienstmann et al., 2015; Kopetz et al., 2015). Therefore, a reliable molecular marker is urgently needed to identify high-risk groups of patients with early-stage CRC and to optimize treatment strategies.

N6-Methyladenosine (m6A)—which is present in messenger RNAs (mRNAs), non-coding RNAs (lncRNAs), and microRNAs (miRNAs) in most eukaryotes—is the most common epigenetic methylation modification of mammalian RNA (Desrosiers et al., 1974; Molinie and Giallourakis, 2017). M6A modification is regulated by a series of protein factors, including methyltransferases (writers), signal transducers (readers), and demethylases (erasers) (Yang et al., 2018a). Abnormal modification of m6A plays an important role in the occurrence and development of many tumors, such as hepatocellular carcinoma, breast cancer, glioblastoma, and lung cancer (Lin et al., 2016; Cui et al., 2017; Vu et al., 2017; Cai et al., 2018; Liu et al., 2019). Moreover, Zhang et al. demonstrated the effect of mutation of the m6A regulator on CRC prognosis (Zhang et al., 2020). Similar to mRNA, lncRNA is also regulated by m6A. Moreover, m6A-related lncRNAs also regulate a series of biological and pathological processes. Recent studies have demonstrated that m6A-related lncRNAs can reliably predict the prognosis of low-grade glioma, lung adenocarcinoma, and gastric cancer (Tu et al., 2020; Wang et al., 2021a; Wang et al., 2021b). Luo et al.(Zuo et al., 2021) further observed that m6A-related lncRNAs were associated with the occurrence and development of CRC, indicating that they may be an accurate prognostic factor for early-stage CRC.

The tumor microenvironment (TME) also plays an important role in tumor initiation and progression, and is comprised of tumor cells, stromal cells and innate and adaptive immune cells (Colangelo et al., 2017). Immunotherapy has quickly become the main treatment modality for CRC, such as programmed cell death1 (PD1)-blocking antibodies (Ganesh et al., 2019). Recently, more studies have focused on comprehensive analysis of specific m6A regulatory factors to strengthen the in-depth understanding of the heterogeneity and complexity of TME (Yang et al., 2019; Li et al., 2020). For example, Na Li et al. (Li et al., 2020) have found a deletion in Alkbh5 that sensitizes tumors to cancer immunotherapy by regulating myeloid-derived suppressor cells and suppressive lymphocyte Treg accumulation. However, at present, there are no studies on this aspect of early-stage CRC.

In the present study, we identified two molecular subgroups of early-stage CRC with unique immune characteristics and showed the potential role of m6A-related lncRNA signatures in predicting prognosis in patients with early-stage CRC.

## Materials and Methods

### Study Approval and Consent

This study was approved by the Medical Ethics Committee of the Sixth Affiliated Hospital of Sun Yat-sen University, Guangzhou, China (no. 2021ZSLYEC-006).

### Data Acquisition and Preprocessing

Initially, we downloaded RNA sequencing data (FPKM value) related to gene expression and the corresponding clinical information for CRC from The Cancer Genome Atlas (TCGA) website (https://portal.gdc.cancer. gov). According to the 8th edition of the American Joint Committee on Cancer, we identified patients with early-stage CRC. The clinical information included age, sex, TNM stage, and survival status. In addition, patients with no survival information or a survival time of less than 3 months were excluded from further evaluation to reduce statistical bias. To distinguish the mRNAs and lncRNAs, we downloaded the GTF files from Ensembl (http://asia.ensembl.org) for further analysis. Data were normalized, processed, and analyzed using R software 4.0.3.

### Identification of m6A-Related lncRNAs

We extracted expression data for 23 m6A-related genes identified in previous studies, including methyltransferases (*METTL3*, *METTL14*, *METTL16*, *WTAP*, *VIRMA*, *ZC3H13*, *RBM15*, and *RBM15B*), demethylases (*ALKBH5* and *FTO*), and recognition proteins (*YTHDC1*, *YTHDC2*, *YTHDF1*, *YTHDF2*, *YTHDF3*, *HNRNPC*, *FMR1*, *LRPPRC*, *HNRNPA2B1*, *IGFBP1*, *IGFBP2*, *IGFBP3*, and *RBMX*) (Arguello et al., 2017; Huang et al., 2021a). Pearson correlation analysis was performed between the m6A genes and all lncRNAs. Absolute correlation coefficients >0.4 and *p* values <0.001 were used to define m6A-related lncRNAs. Univariate Cox regression analysis was used to screen for m6A-related lncRNAs significantly associated with overall survival (OS) (*p* < 0.01).

### Unsupervised Clustering of Seven m6A-Related lncRNAs Associated With Prognosis

Unsupervised clustering analysis was used to classify patients with early-stage CRC into different subgroups based on the expression of the seven m6A-related lncRNAs associated with prognosis. The ConsensusClusterPlus package was used to perform the above steps and was repeated 1,000 times to ensure the stability of the classification (Wilkerson and Hayes, 2010). Then, we used the Euclicean disease to compute the similarity disease between patients with early-stage CRC. The optimal number of clusters was identified by CDF and consensus matrices.

GSEA (version 4.1.0) software was applied to determine the gene expression in cluster1 and cluster2 in the Molecular Signatures database (MSigDB) Collection (c2.cp.kegg. v7.2symbols.gmt) to further analyze the difference in KEGG pathway enrichment. The threshold of statistical significance was defined by a nominal *p*-value <0.05 and a false discovery rate (FDR) *q* value <0.25 (Subramanian et al., 2005).

### Immune Characteristics for Molecular Subtypes of Early-Stage CRC

The ESTIMATE algorithm was used to calculate the ESTIMATE score, stromal score, and immune score between cluster 1 and cluster 2 (Gentles et al., 2015). The CIBERSORT algorithm, which can sensitively and specifically distinguish 22 human immune cell phenotypes, was used to quantify the immune cells in early-stage CRC samples. The Wilcoxon signed-rank test was used to analyze the differences in immune infiltrating cells between the different molecular subtypes using the above method. In addition, levels of checkpoint gene expression were also compared. Furthermore, the relationship between PD1 and m6A-related lncRNAs associated with prognosis was quantified using Pearson correlation analysis. The thresholds were set as *p*-value < 0.05.

### Establishment and Validation of the m6A-Related lncRNA Risk Prognosis Model

First, patients with early-stage CRC were randomly divided into training and test cohorts at a ratio of 1:1 using the caret package. Second, least absolute shrinkage and selection operator (LASSO) Cox regression analysis was performed to analyze the best candidates and multiple m6A-related lncRNA characteristics for constructing the prognostic signature. Ten-fold cross-validation was used to prevent overfitting. Based on the above model, the risk score for each patient was calculated as follows: risk score = $\overset{k}{\underset{i=1}{\sum }}\beta iSi$ (*k*: the number of m6A-related lncRNAs incorporated into the signature; *βi*: the coefficient for each m6A-related lncRNA; *Si*: the level of m6A-related lncRNA expression). Then, patients with early-stage CRC were divided into high-risk and low-risk groups based on the median score of the training cohort. The Kaplan–Meier method and log-rank test were used to evaluate the survival difference between the two risk groups in the training and test cohorts. The area under the curve (AUC) and time-dependent receiver operating characteristic (ROC) curves were examined to assess the predictive power of the signal for survival using the “survival” and “survivalROC” packages in R software. The “survcomp” package is used to calculate the Consistency index (C-index) to compare the prediction accuracy of prognostic features. Univariate and multivariate Cox regression analyses were conducted to verify the independence of the risk score by comparing common clinical features including age, sex, and stage. We used a forest map to demonstrate the results. The R package used in the above operation is “survival”, “pheatmap”, and “ggpupr”.

### Construction of a Predictive Nomogram for OS in Patients With Early-Stage CRC

Nomograms are widely used to predict the outcomes of patients with cancer (Iasonos et al., 2008). Risk scores and clinical indicators were incorporated into a nomogram to evaluate the probability of 1, 2, and 3 years OS in patients with early-stage CRC using “rms” R package. The C-index, ROC curves, and calibration curves (by a bootstrap method with 1,000 peplicates) were used to evaluate the predictive ability and discriminative value of the nomogram.

### Chemosensitivity Prediction

The Cancer Drug Sensitivity Genomics (GDSC) database (https://cancerrxgene.org) can be used for large-scale drug screening. Combined with genomic analysis, the response of tumors to chemotherapy drugs can be systematically identified. Using the GDSCdatabase, the half-maximal inhibitory concentration (IC_50_) of common chemotherapeutic drugs for gastrointestinal tumors (camptothecin, cisplatin, rapamycin, bryostatin, and methotrexate) was calculated to evaluate the clinical application of this model in the treatment of early-stage CRC. The R package used in the above operation is “pRRophetic”. Then, we compared the difference in the IC_50_ between the high-risk group and low-risk group using the Wilcoxon signed-rank test. To visualize the data, box drawings were created using “pRRophetic” and “ggplot2” in R (Slavin, 1989).

### Statistical Analysis

R software (v 4.0.3) was used for statistical analysis (R packages: “limma,” “pheatmap,” “survival,” “survminer,” “glmnet,” “reshape2,” “ggpubr,” “ConsensusClusterPlus,” “ggplot2,” “corrplot,” “utils,” “vioplot,” “plyr,” “grid,” “gridExtra (multi-GSEA),” “pRRophetic,” “caret,” “glmnet,” “timeROC,” “ggExtra”, “survcomp”). In the above statistical tests, a two-sided *p*-value <0.05 was considered statistically significant.

## Results

### Identification of m6A-Related lncRNAs Associated With Prognosis in Early-Stage CRC

**FIGURE 1:**
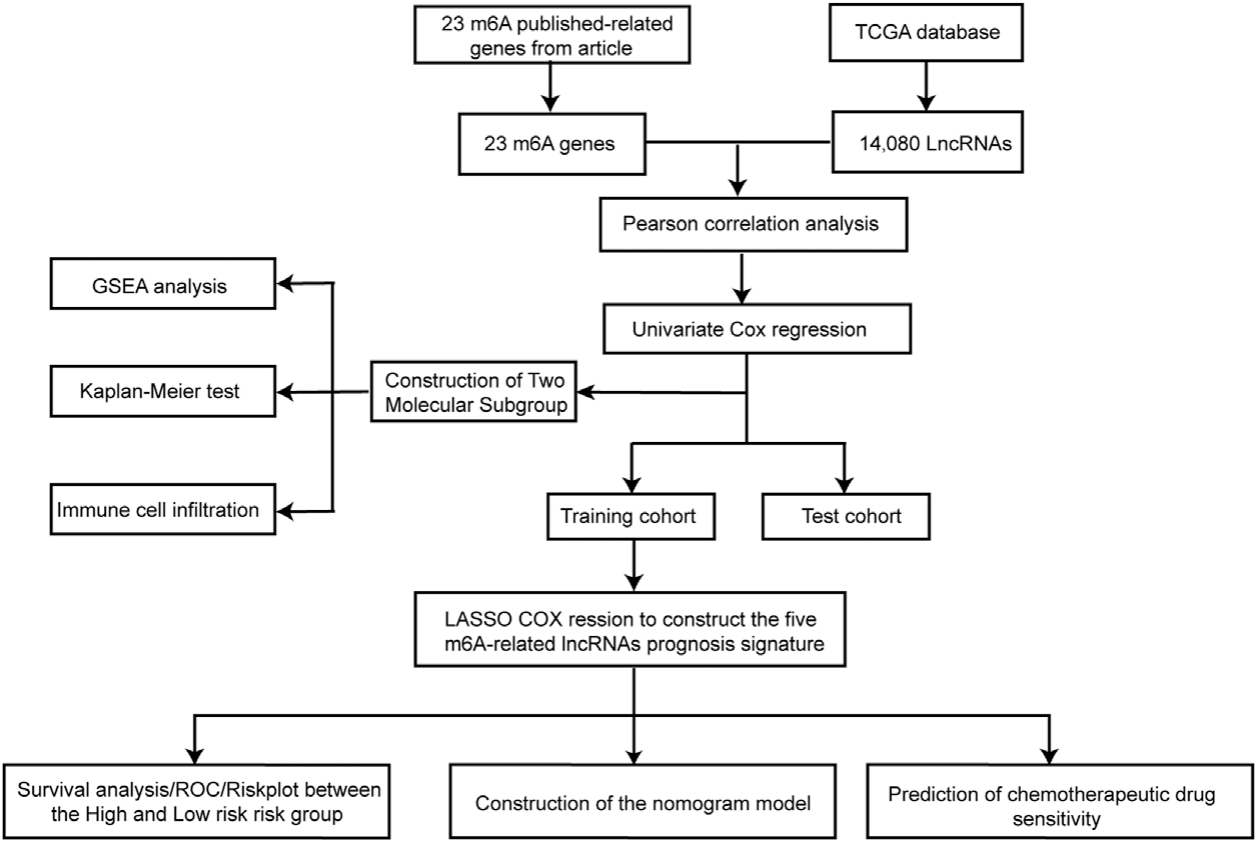
Flow diagram of the study.

**FIGURE 2:**
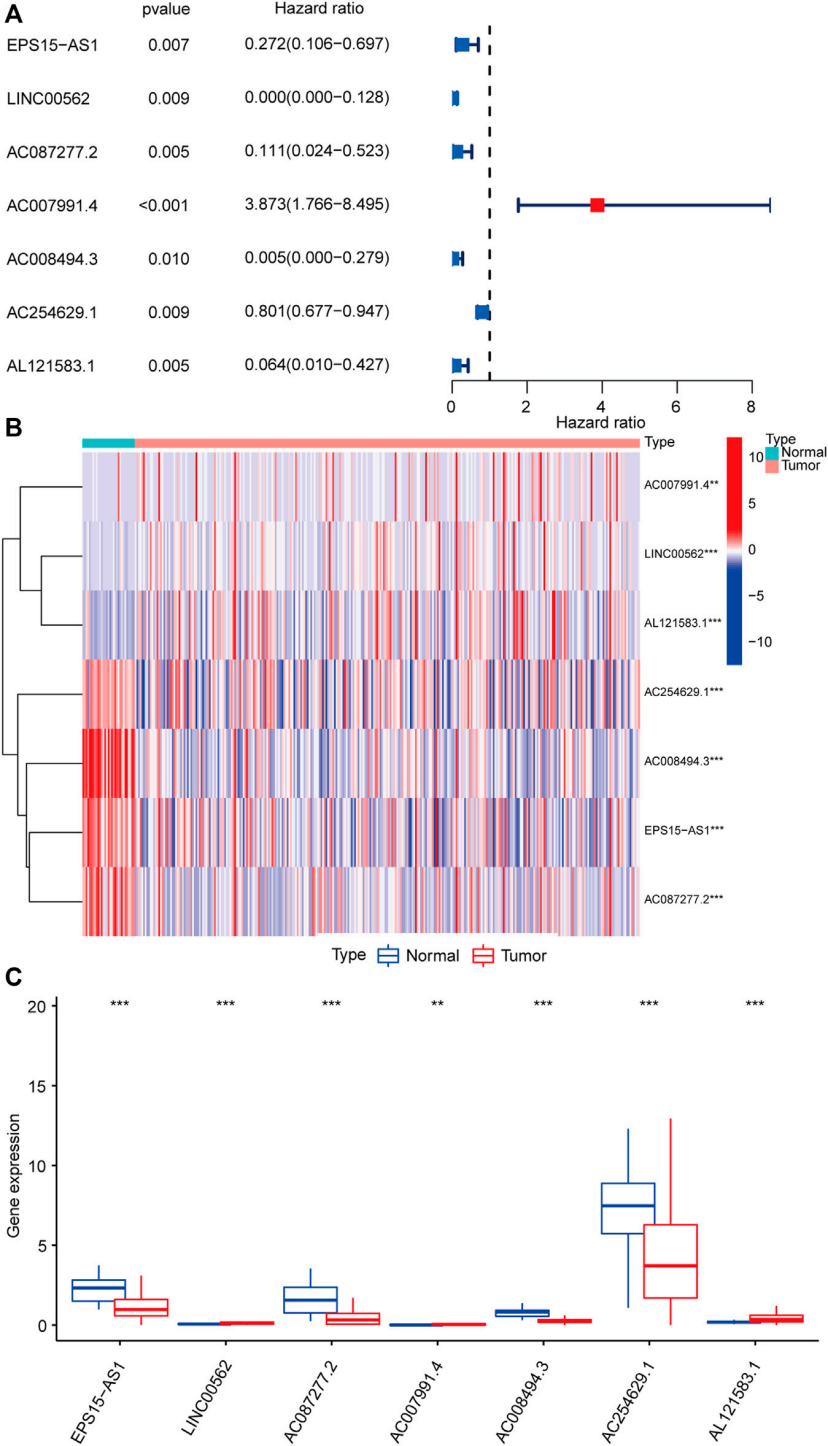
Identification of m6A-related lncRNAs associated with the prognosis of early-stage CRC. Forest plot of seven m6A-related lncRNAs associated with prognosis based on univariate Cox regression analysis. Red indicated that the m6a-related lncRNA is a risk factor for the prognosis of patients with early-stage CRC, and blue indicated that m6a-related lncRNA is a protective factor for the prognosis of patients with early-stage CRC **(A)**. Heatmap of seven differentially expressed m6A-related lncRNAs between normal colorectal tissue (marked in green) and early-stage CRC (marked in red) **(B)**. Expression patterns of seven m6A-related lncRNAs in normal tissue (marked in blue) and tumor tissue (marked in red) **(C)**. *p < 0.05, **p < 0.01*,* and ***p < 0.001. Abbreviation: m6A-related lncRNAs, N6-methyladenosine-related long non-coding RNAs; CRC, colorectal cancer.

The bioinformatic analysis of this study was performed as depicted in Figure 1. First, a total of 14,081 lncRNA expression and 23 m6A-related gene expression profiles were obtained from 299 early-stage CRC and 31 normal samples. The corresponding clinical data of 251 patients were also extracted from TCGA (Table 1). Then, 1,478 m6A-related lncRNAs were identified and seven survival-associated m6A-related lncRNAs were obtained (Figure 2A). The expression levels of these lncRNAs differed significantly between normal and tumor tissues (*p* < 0.05, Figures 2B,C). Expression levels of the three m6A-related lncRNAs (LINC00562, AC007991.4, and AL121583.1) in tumor tissue were significantly higher than those in normal tissues, whereas the expression levels of the remaining four lncRNAs (EPS15-AS1, AC087277.2, AC008494.3, and AC244629.1) were higher in normal tissues.

**TABLE 1:** Clinical characteristics of early-stage CRC in TCGA.

Clinical characteristics	Cases
Patients, n	251
Survival status, n (%)	
Alive	228(90.8%)
Dead	23(9.2%)
Age, n (%)	
≤60	69(27.4%)
>60	182(72.6%)
Sex, n (%)	
Male	144(57.4%)
Female	107(42.6%)
TNM stage, n (%)	
Stage Ⅰ	83(33.1%)
Stage Ⅱ	168(66.9%)

CRC, colorectal cancer; TCGA, The Cancer Genome Atlas.

### Construction of Two Molecular Subgroups of Early-Stage CRC Using Seven Survival-Associated m6A-Related lncRNAs

**FIGURE 3:**
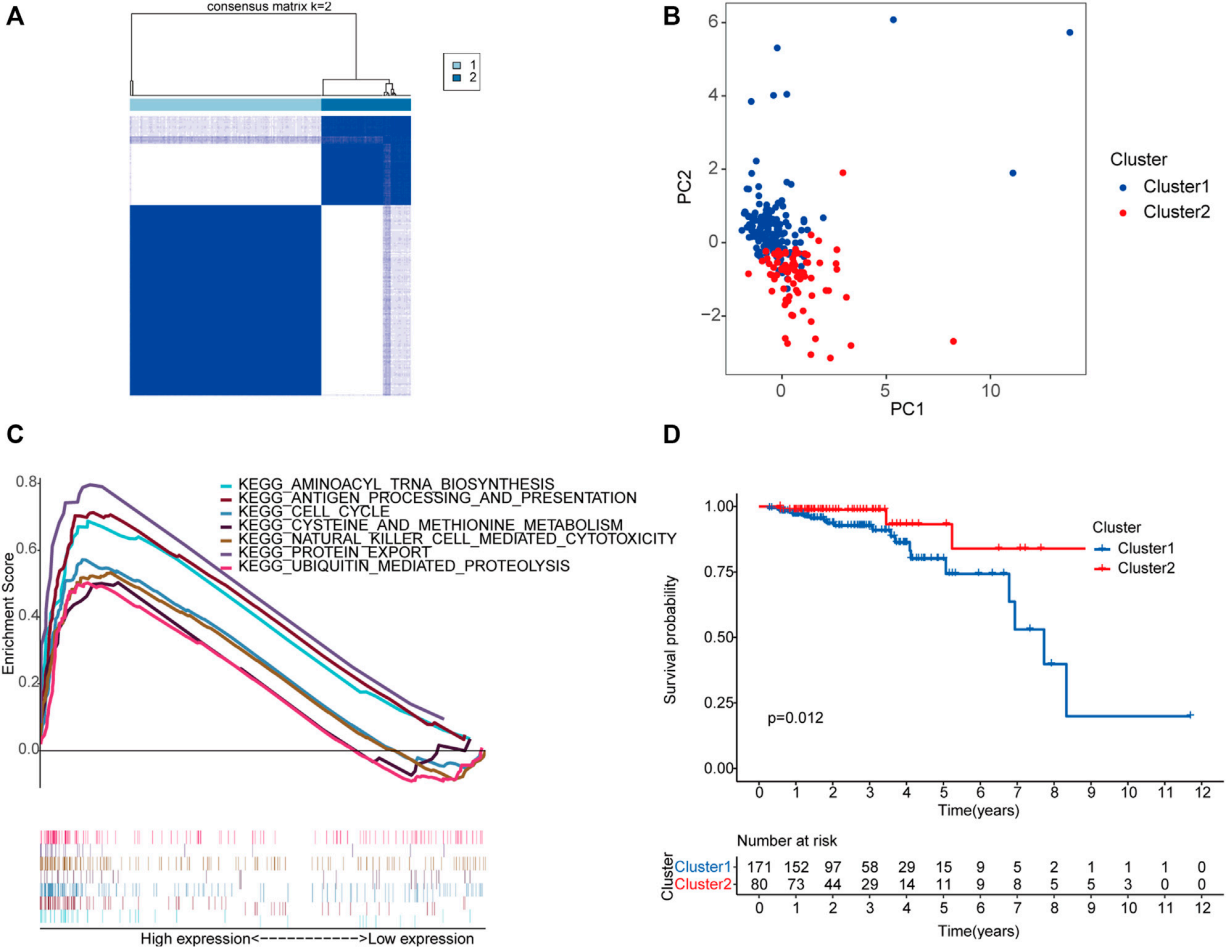
Construction of two molecular subgroups of early-stage CRC using seven m6A-related lncRNAs associated with prognosis. The consensus matrices of the TCGA database for *k* = 2 **(A)**. PCA between cluster one (marked in blue) and cluster two (marked in red) **(B)**. GSEA analysis of the different activation status of the biological pathways between cluster one and cluster 2 **(C)**. Survival analysis of two subgroups (blue represented cluster 1, and red represented cluster 2) based on data from 251 patients with early-stage CRC in the TCGA cohort **(D)**. Abbreviation: CRC, colorectal cancer; m6A-related lncRNAs, N6-methyladenosine-related long non-coding RNAs; PCA, principal component analysis; GSEA, gene set enrichment analysis; TCGA, The Cancer Genome Atlas.

Unsupervised clustering identified two molecular subgroups as the optimal number of clusters, including 171 cases in cluster one and 80 cases in cluster two (Figure 3A, Supplementary Figure S1). Principal component analysis (PCA) was conducted based on the above classification methods, and there was a significant difference in the distributions of cluster one and cluster two (Figure 3B). The KEGG results revealed that “aminoacyl tRNA biosynthesis,” “antigen processing and presentation,” “cell cycle,” “cysteine and methionine metabolism,” “natural killer cell-mediated cytotoxicity,” “protein export,” and “ubiquitin-mediated proteolysis” were markedly enriched in cluster 1 (Figure 3C). Furthermore, the prognosis of cluster 1 was poorer than that of cluster 2 (Figure 3D). These results indicate that there are molecular subgroups of early-stage CRC with different characteristics.

### Immune Characteristics of the Two Molecular Subgroups of Early-Stage CRC

**FIGURE 4:**
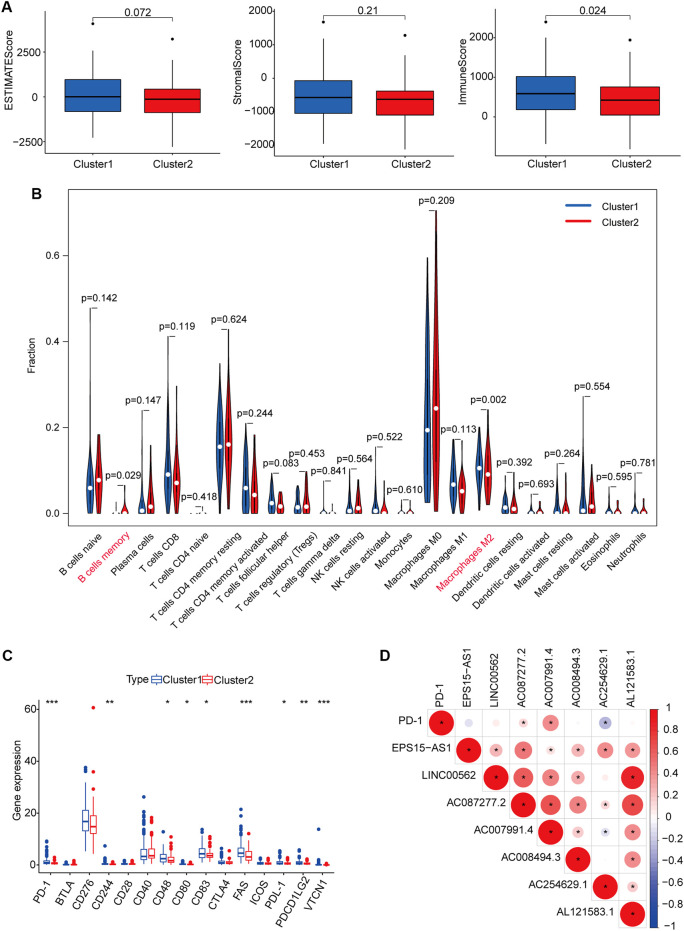
Immune cell infiltration and biological functions in the two subgroups. ESTIMATE, stromal, and immune scores in cluster 1 (marked in blue) and cluster 2 (marked in red) **(A)**. Abundance of 22 immune cells in the two subgroups (blue represented cluster 1, and red represented cluster 2) **(B)**. Expression of checkpoint genes in the two subgroups (Blue represented cluster 1, and red represented cluster 2). *p < 0.05, **p < 0.01, and ***p < 0.001 **(C)**. Heatmap for the correlation between PDL1 gene and seven prognostic m6A-related lncRNAs. Blue represented a positive correlation between the PD-1 gene and the m6A-related lncRNA, and red represented a negative correlation between the PD-1 gene and the m6A-related lncRNA. The darker the color, the stronger the relevance **(D)**. *p < 0.05. Abbreviation: m6A-related lncRNAs, N6-methyladenosine-related long non-coding RNAs.

In addition to tumor cells, the tumor microenvironment includes immune cells and stromal cells, which provide protection and support for the occurrence and development of tumors. As shown in Figure 4A, no significant differences in ESTIMATE or stromal scores were observed between cluster 1 and cluster 2; however, immune scores were higher in cluster 1 than in cluster two (*p* = 0.024), indicating that levels of immune cells differ based on molecular subgroup. Our analysis revealed enrichment of the proportion of M2 macrophages in cluster one, as well as a higher proportion of memory B cells in cluster two (Figure 4B). We also examined differences in the expression profiles of immune checkpoint genes between clusters 1 and 2. Levels of most immune checkpoint genes were higher in cluster 1 than in cluster two, suggesting that patients in cluster 1 were more likely to exhibit immune escape and poor prognosis (Figure 4C). We also observed that the expression of PD1 was positively correlated with AC087277.2 and AC007991.4 yet negatively correlated with AC254629.1 (Figure 4D).

### Construction and Validation of the m6A-Related lncRNA Prognostic Signature

**FIGURE 5:**
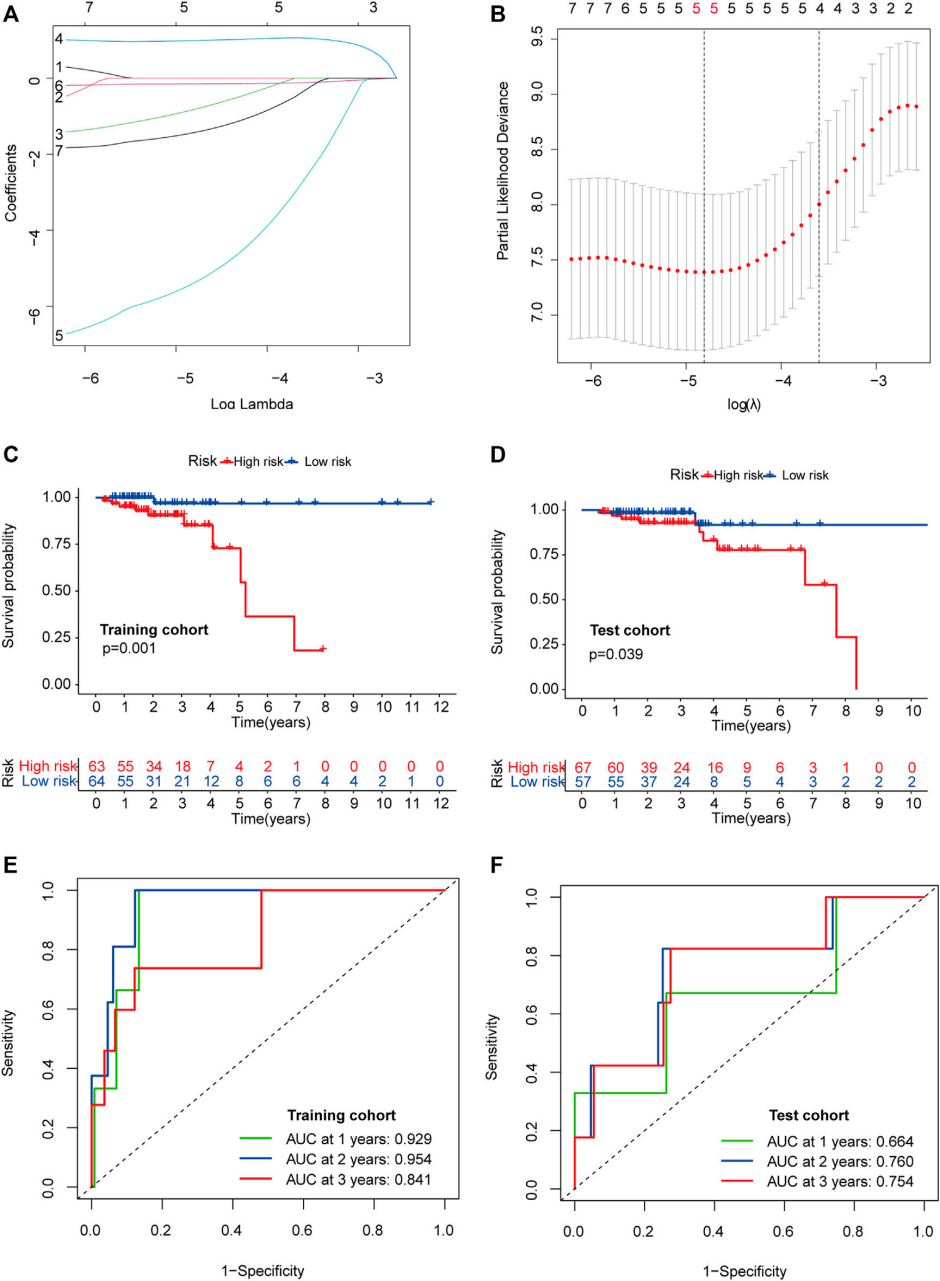
Construction and validation of the prognostic risk model. LASSO coefficient profiles of seven candidates in the training cohort **(A)**. Selection of the optimal parameter (lambda) in the LASSO model **(B)**. Survival analysis in the high- (marked in red) and low-risk groups (marked in blue) in the training cohort **(C)** and test cohort **(D)**. Time-dependent ROC analysis for 1, 2, and 3-years OS prediction among patients with early-stage CRC in the training **(E)** and test cohorts **(F)**. Abbreviation: LASSO, least absolute shrinkage and selection operator; ROC, receiver operating characteristic; OS, overall survival; CRC, colorectal cancer.

**FIGURE 6:**
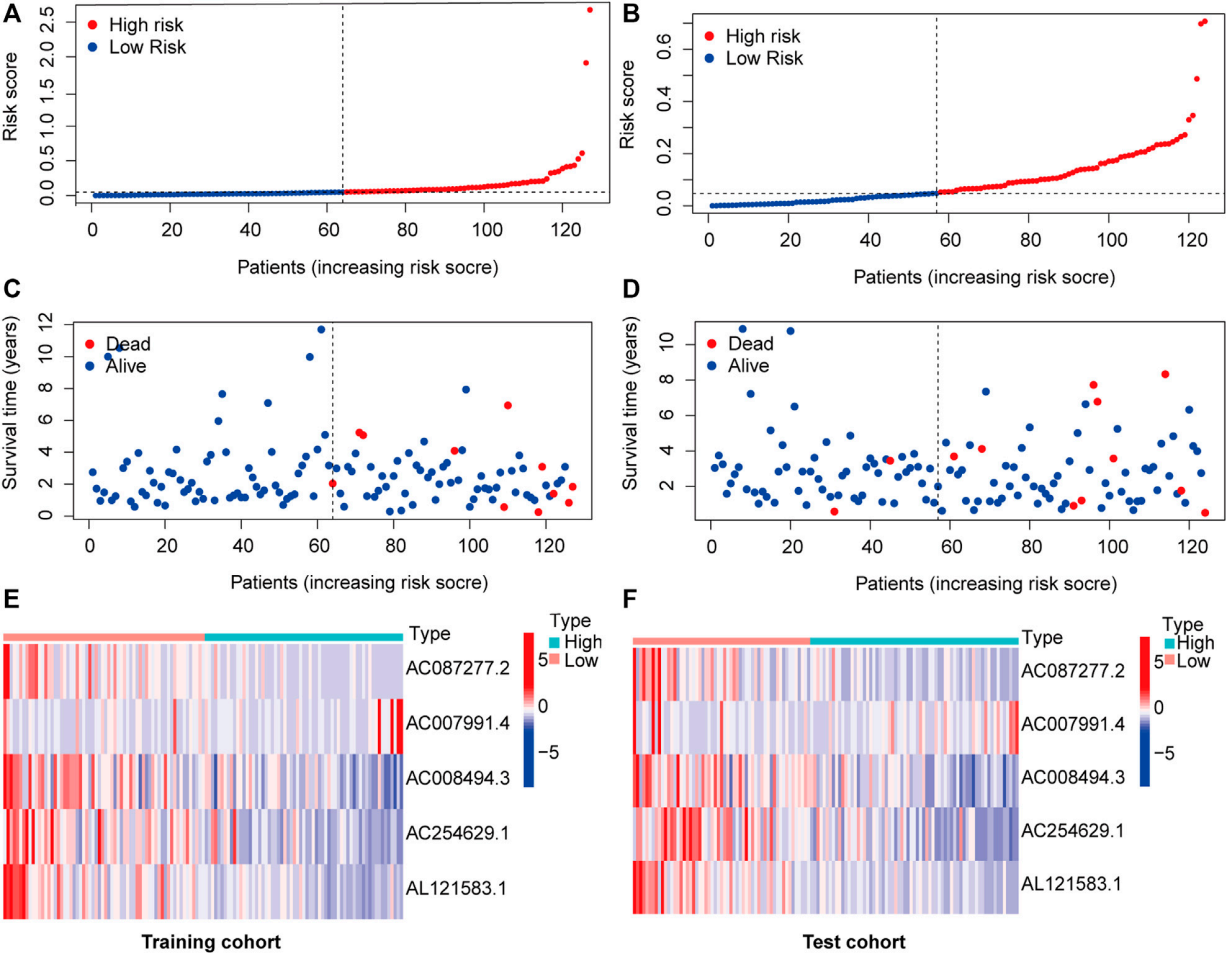
m6A-related lncRNA signature risk score analysis. Risk curves of each sample reordered by risk scores in the training cohort **(A)** and test cohort **(B)**. Living status of patients with early-stage CRC in the training cohort **(C)** and test cohort **(D)**. Heatmap of five m6A-related lncRNA expression profiles in the low- (marked in red) and high-risk groups (marked in green) in the training cohort **(D)** and test cohort **(F)**. Abbreviation: m6A-related lncRNAs, N6-methyladenosine-related long non-coding RNAs; CRC, colorectal cancer.

**FIGURE 7:**
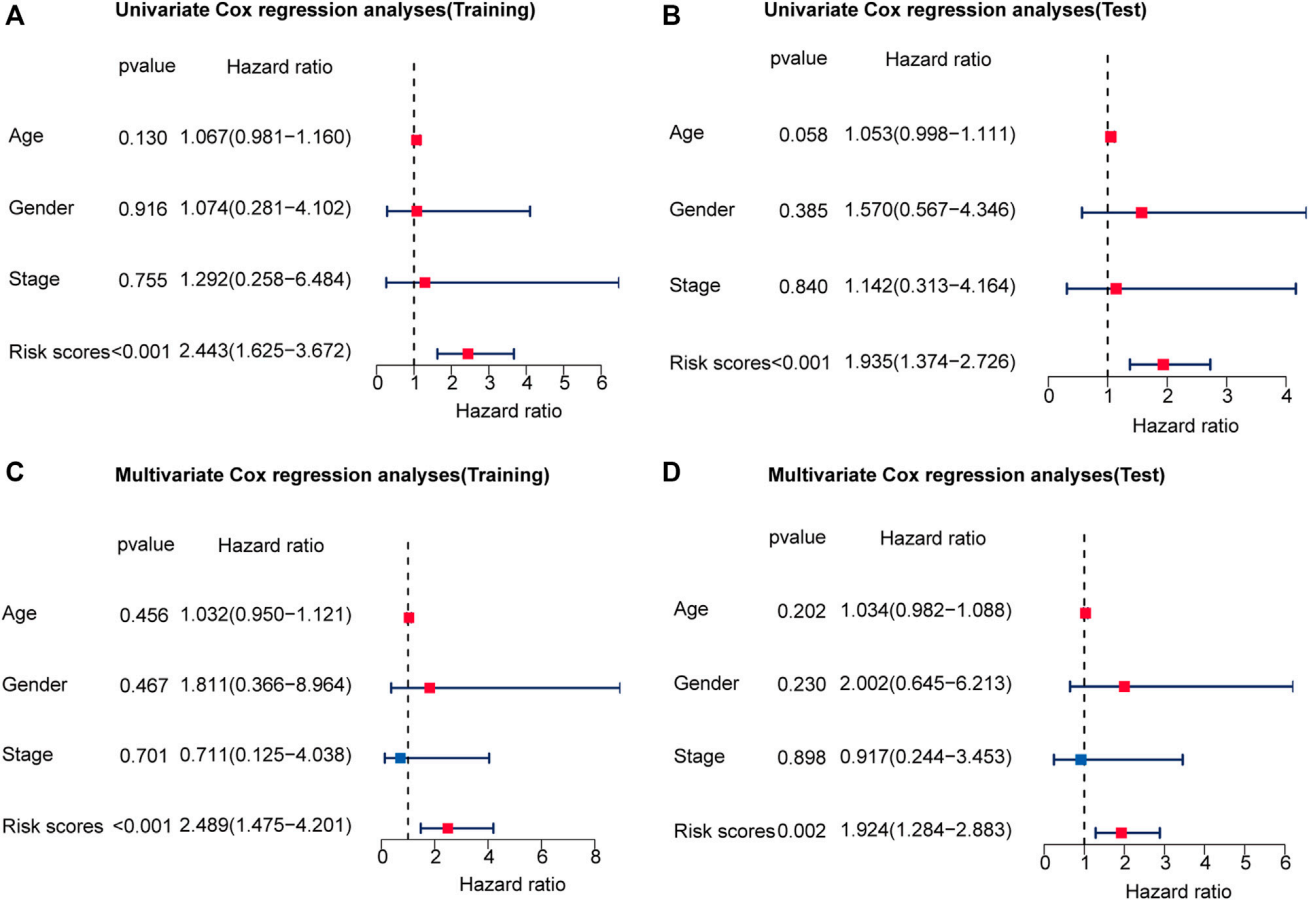
The risk score is an independent prognostic factor for early-stage CRC. Univariate regression analysis of early-stage CRC in the training cohort **(A)** and test cohort **(B)**. Multivariate Cox regression analysis of early-stage CRC in the training cohort **(C)** and test cohort **(D)**. Red indicated that the indicator is a risk factor for the prognosis of patients with early-stage CRC, and blue indicated that the indicator is a protective factor for patients with early-stage CRC. And *p*-value < 0.05 was considered statistically significant.

The 251 patients were randomly divided into a training cohort (*n* = 127) and a test cohort (*n* = 124) in a 1:1 ratio. To build the m6A-related lncRNA signature for forecasting the prognosis of patients with early-stage CRC, LASSO Cox analysis was performed based on the seven m6A-related lncRNAs in the training cohort. The m6A-related lncRNA signature included five m6A-related lncRNAs and the coefficients of each (Figures 5A,B). Based on the coefficients, the risk scores of each patient were calculated using the following formula: risk score = –0.828 × AC087277.2 + 0.989 × AC007991.4–5.396 × AC008494.3–0.151 × AC254629.1–1.435 × AL121583.1. Patients in the training cohort were grouped into low-risk and high-risk groups according to the median value of the risk scores. The Kaplan–Meier survival curve showed that patients with early-stage CRC with higher risk scores had poorer clinical outcomes than those with relatively lower risk scores (Figure 5C). The ROC curve showed that the m6A-related lncRNA signature had a good ability to predict OS in the training cohort (1 year AUC = 0.929, 2-years AUC = 0.954, 3-years AUC = 0.841; Figure 5E). Consistent with the results in the training cohort, higher risk scores were associated with shorter OS times and lower OS rates in the test cohort (Figure 5D). The ROC analysis also revealed that the m6A-related lncRNA signature had a strong prognostic value in the test cohort (1 year AUC = 0.664, 2 years AUC = 0.760, 3 years AUC = 0.754; Figure 5F). The distributions of risk, survival status, and expression of five m6A-related lncRNAs in the training and test cohorts are shown in Figure 6, which suggests that patients with higher risk scores exhibited shorter OS. Univariate and multivariate Cox regression analyses show that risk scores were significantly related to OS, independent of clinical parameters (Figures 7A–D).

### Construction of the Nomogram Based on the m6A-Related lncRNA Signature

**FIGURE 8:**
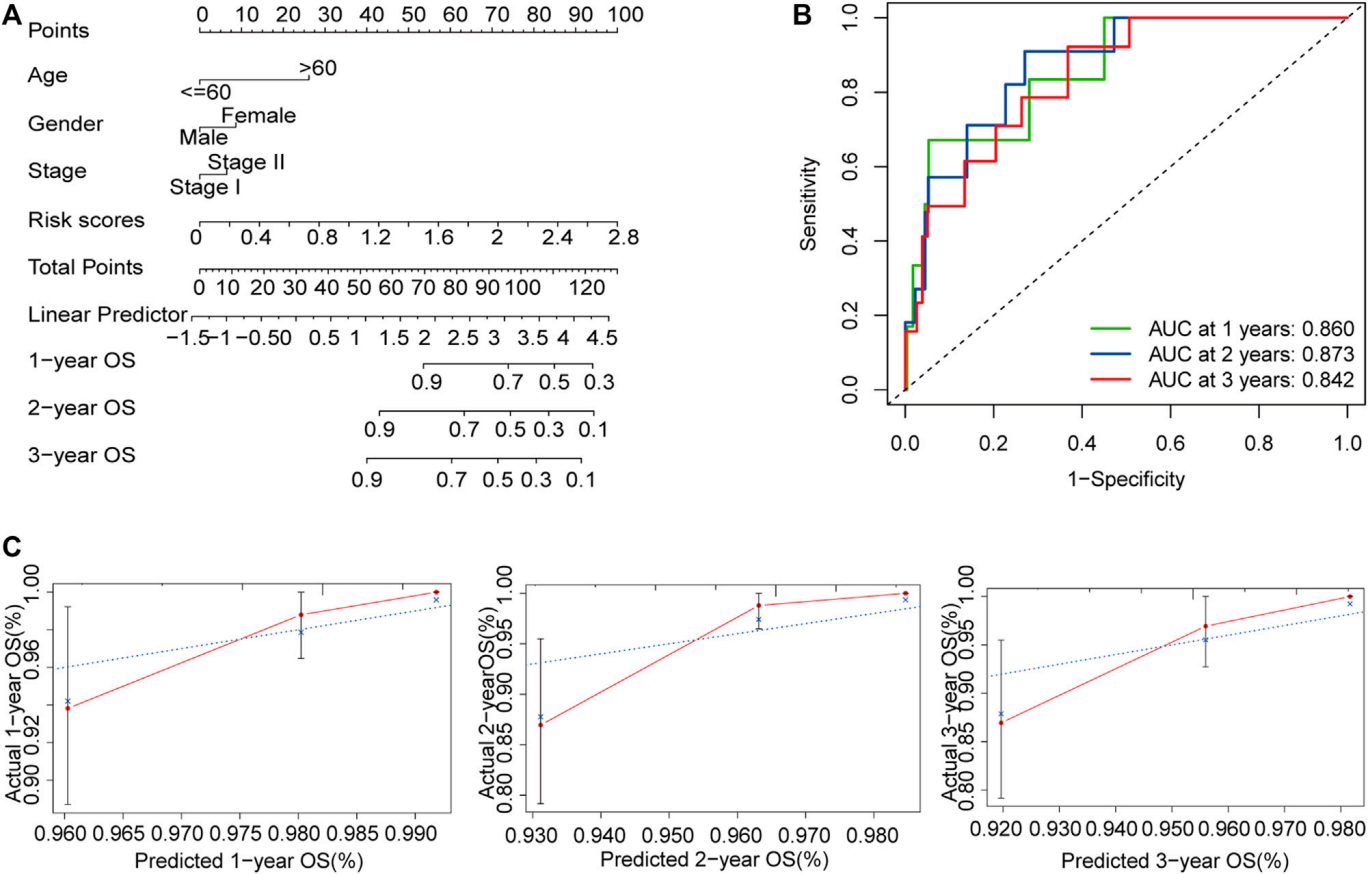
Construction of a nomogram for the prediction of outcomes in patients with early-stage CRC. The nomogram combining clinical features and the m6A-related lncRNA signature **(A)**. ROC curves for the nomogram for predicting 1, 2, and 3-years OS **(B)**. Calibration plot showing that the predicted probability and the actual probability were consistent **(C)**. Abbreviation: m6A-related lncRNAs, N6-methyladenosine-related long non-coding RNAs; CRC, colorectal cancer; ROC, receiver operating characteristic; OS, overall survival.

To develop a clinically applicable method for predicting OS in patients with early-stage CRC, we built a nomogram using age, sex, stage, and risk scores (Figure 8A). The C-index of the nomogram for predicting the OS rate was 0.821. The nomogram had excellent accuracy regarding the 1, 2, and 3-years OS rates (AUC = 0.860, 0.873, and 0.842, respectively; Figure 8B). Moreover, the calibration curve demonstrated that the nomogram was suitable for clinical practice (Figure 8C).

### Response to Chemotherapy in the High and Low-Risk Groups

**FIGURE 9:**
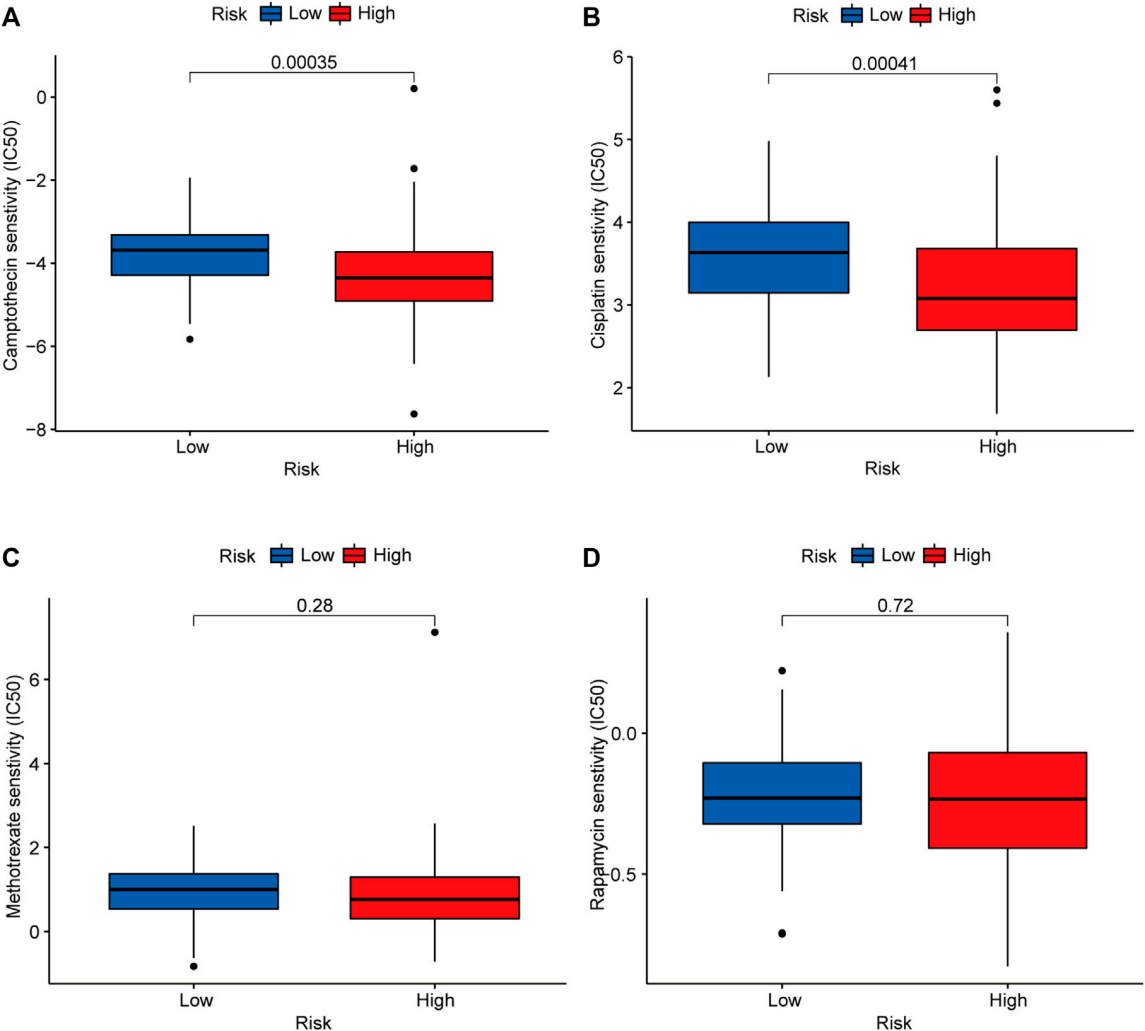
Candidate chemotherapeutic drugs targeting the m6A-related lncRNA signature. IC50s of camptothecin **(A)**, cisplatin **(B)**, methotrexate **(C)**, and rapamycin **(D)** between the low- (marked in blue) and high-risk groups (marked in red). Abbreviation: m6A-related lncRNAs, N6-methyladenosine-related long non-coding RNAs.

To identify potential chemotherapeutic drugs targeting the m6A-related lncRNA signature for treating patients with early-stage CRC, the pRRophetic algorithm was used to estimate the therapeutic response based on the IC_50_ for each sample. We found that the high-risk group was more sensitive to camptothecin and cisplatin, which may provide insight into new treatment options for patients with early-stage CRC (Figures 9A–D).

## Discussion

Even after radical surgical treatment, the risk of recurrence in patients with stage I and stage II CRC is still 5 and 20%, respectively, (Dotan and Cohen, 2011). Conventional high-risk characteristics, such as MSI status and bowel obstruction, cannot meet the demands for predicting prognosis in patients with early-stage CRC. However, numerous studies have highlighted the relevance of lncRNAs. Specific lncRNAs can be modified by m6A regulators to participate in tumorigenesis, development, and metastasis (Tu et al., 2020). In the present study, we extracted data regarding the expression of lncRNA and m6A in these patients from TCGA. Seven m6A-associated lncRNAs related to prognosis were identified using univariate Cox regression analysis. Based on these lncRNAs, we identified two molecular subgroups of early-stage CRC with unique immune characteristics. Furthermore, we constructed and validated a novel m6A-related lncRNA model for predicting OS in patients with early-stage CRC, following which we developed a nomogram for clinical application in this patient population. Finally, we assessed responses to common chemotherapeutic drugs in the high- and low-risk groups.

Here, based on seven m6A-related lncRNAs associated with prognosis, we identified two molecular subgroups of early-stage CRC with significantly distinct features. Prognosis was worse for cluster 1 than for cluster two, and cluster 1 was characterized by increased numbers of M2 macrophages, decreased numbers of memory B cells, and increased expression of checkpoint genes when compared with cluster two. Tumor-associated macrophages are comprised of M1 and M2 macrophages (Mantovani et al., 2017). It is well known that M1 (anti-tumor) and M2 (pro-tumor) phenotypes are related to different immunomodulatory functions (Yang et al., 2018b). In a previous study, Catherine et al. inferred that B lymphocytes may exert anti-tumor immune functions by collaborating with T lymphocytes (Sautès-Fridman et al., 2016). Edin et al. also found that tumor-infiltrating CD20^+^ B lymphocytes are associated with a favorable prognosis in CRC (Edin et al., 2019). Thus, cluster 1 may be associated with immunosuppression. Furthermore, expression levels of immune checkpoint genes were higher in cluster 1 than in cluster two, which is a characteristic of immunosuppression. This finding suggests that patients in cluster 1 were more likely to exhibit immune escape and poor prognosis than those in cluster two.

It has been reported that m6A-related lncRNAs are involved in the occurrence and development of a variety of tumors. For example, m6A on lncRNA NEAT1-1 may be a novel specific marker for bone metastasis and is correlated with poor prognosis (Wen et al., 2020). METL3 and METTL14 on LNCAROD have tumor-promoting functions in the development of head and neck squamous cell carcinoma (Ban et al., 2020). Moreover, m6A-related lncRNAs can reliably predict the prognosis in several tumors such as metastatic skin cutaneous melanoma and adrenocortical carcinoma (Huang et al., 2021b; Jin et al., 2021). Similar to these studies, we conducted a series of bioinformatics analyses to generate a new m6A-related lncRNA signature for early-stage CRC prognosis. The signature included AC087277.2, AC007991.4, AC008494.3, AC254629.1, and AL121583.1. The construction of a novel signature allowed us to distinguish samples at different risks. In the training cohort, the OS time of early-stage CRC was shorter in the high-risk group than in the low-risk group, and consistent results were obtained in the test cohort. In addition, univariate and multivariate Cox regression analyses confirmed that the risk score was an independent predictor of OS in patients with early-stage CRC. Taken together, these results highlight the ability of our m6A-related lncRNA prognostic signature to accurately predict OS in this population.

High-risk patients with early-stage CRC are usually treated via a combination of surgery and chemotherapy. Using the GDSC database, we found that high-risk patients were more sensitive to commonly used chemotherapy drugs (including camptothecin and cisplatin) than low-risk patients, which may provide new treatment options for patients with early-stage CRC. Camptothecin is an alkaloid extracted from *Camptotheca acuminata* that can inhibit the catalytic activity of topoisomerase (Hsiang et al., 1985; Mollica et al., 2012). In agreement with previous reports (Cross-Knorr et al., 2013), camptithecin can improve patients with early-stage CRC prognosis by inhibiting PKIP phosphorylation and STAT3 activation. Cisplatin is one of the main chemotherapy drugs used by oncologists to treat CRC (AlShamaileh et al., 2017). It has antitumor effects by binding to DNA and inducing DNA damage (Huang et al., 2019). More clinical trials are needed to explore the applicability of these two chemotherapeutic agents in patients with early-stage CRC.

However, our study had several limitations. First, this study was based on the TCGA database, and there was no external data set for verification. When searching for other data sets, such as the Gene Expression Omnibus, we were unable to locate one with both clinical information and the corresponding lncRNA and mRNA expression data. Future studies should use other data sets to verify the performance of our prognostic signature. Second, our findings must be verified *in vivo* and *in vitro*. Lastly, we did not explore the underlying mechanism of the studied m6A-related lncRNAs. Therefore, functional studies should examine these five lncRNAs individually and in combination to further verify the prediction accuracy of the signature and discover potential regulatory mechanisms. Despite these limitations, to the best of our knowledge, this is the first study to report an established external verification of m6A-related lncRNA signatures for early-stage CRC.

## Conclusion

In summary, our analysis of seven m6A-related lncRNAs associated with prognosis identified two molecular subgroups of early-stage CRC with unique immune features. Subsequent analyses demonstrated the usefulness of a five m6A-related lncRNA signature as a potential indicator of prognosis in patients with early-stage CRC. Further studies are required to explore the mechanisms underlying the association between the significant m6A-related lncRNAs and CRC prognosis and to verify the applicability of our prognostic signature.

## Data Availability

The original contributions presented in the study are included in the article/Supplementary Material further inquiries can be directed to the corresponding author.

## Abbreviations

CRC, colorectal cancer; GSEA, gene set enrichment analysis; IC50, half-maximal inhibitory concentration; LASSO, least absolute shrinkage and selection operator; lncRNA, non-coding RNA; m6A­related lncRNAs, N6-methyladenosine-related long non-coding RNAs; m6A, N6-methyladenosine; miRNA, microRNAs; mRNAs, messenger RNAs; MSI, microsatellite instability; OS, overall survival; PD1, programmed cell death protein 1; ROC, receiver operating characteristic curve; TCGA, The Cancer Genome Atlas.
