# Supplementary material for: Prognostic Value of N6-Methyladenosine-Related lncRNAs in Early-Stage Colorectal Cancer: Association With Immune Cell Infiltration and Chemotherapeutic Drug Sensitivity
Source: Front Mol Biosci. 2021 Oct 12;8:724889. doi: 10.3389/fmolb.2021.724889 (PMC8546174; doi:10.3389/fmolb.2021.724889)
Supplement: Supplementary file 1 [file DataSheet1.docx]

Supplementary Material

# Supplementary Figures and Tables

## Supplementary Figures


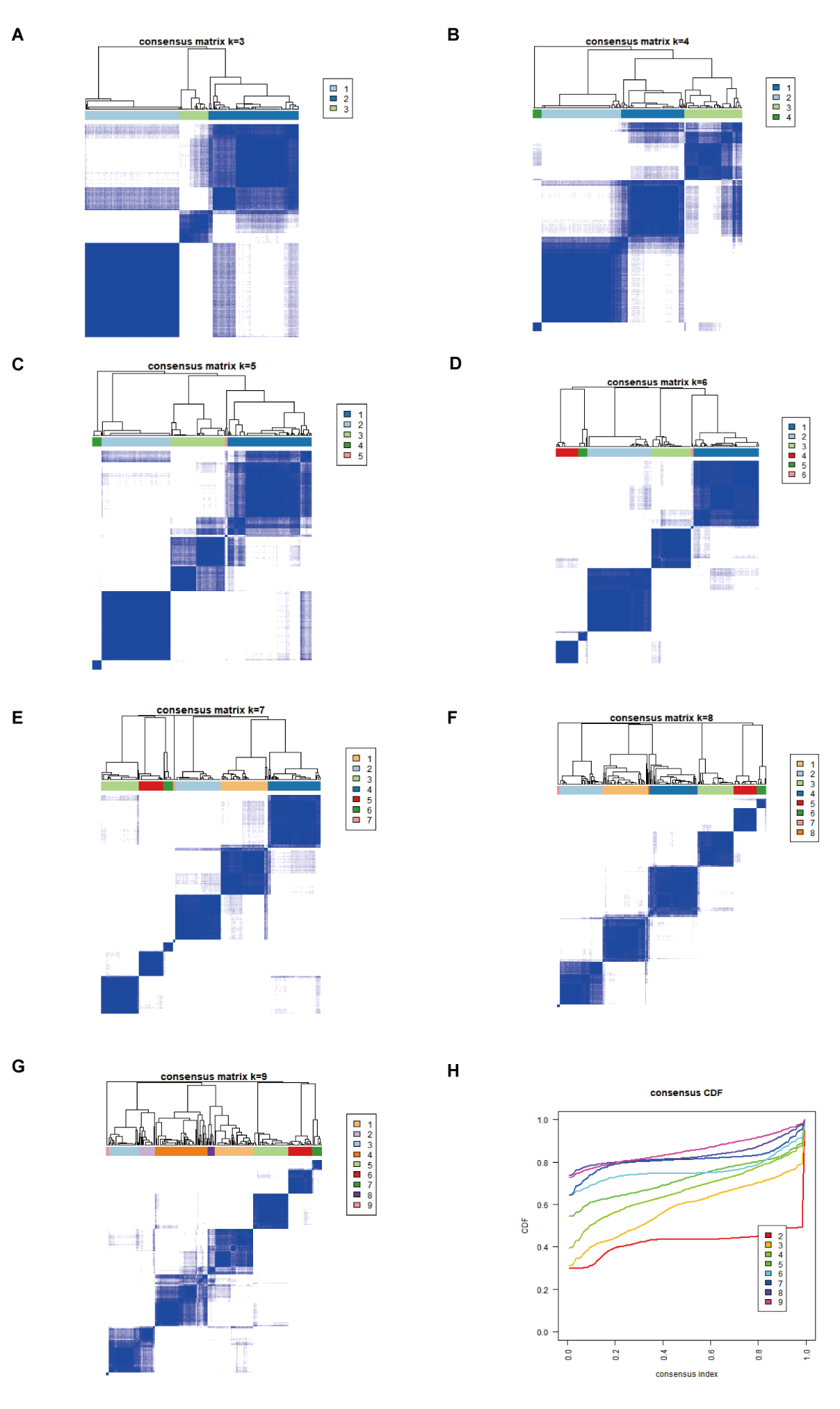


**Supplementary Figure 1.** Consensus clustering of m6A-related lncRNAs in patients with early-stage CRC. TCGA database consensus matrices for k=2 **(A-G)**. CDF curves in different consensus cluster (K=2-9) analyses**(H)**. Abbreviation: m6A-related lncRNAs: N6-methylandenosine-related long non-coding RNAs; CRC: colorectal cancer; CDF: cumulative function .
